# Validation and adaptation of a Turkish version of the dietarian identity questionnaire

**DOI:** 10.1371/journal.pone.0327116

**Published:** 2025-06-25

**Authors:** Damla Gumus, Arife Macit, Senol Demirci, Mevlude Kizil

**Affiliations:** 1 Department of Nutrition and Dietetics, Faculty of Health Sciences, Hacettepe University, Sihhiye, Ankara, Türkiye; 2 Department of Nutrition and Dietetics, Faculty of Health Sciences, Munzur University, Tunceli, Türkiye; 3 Türkiye Health Care Quality and Accreditation Institute (TUSKA), Ankara, Türkiye; The University of Sydney, AUSTRALIA

## Abstract

Dietarian identity reflects an individual’s cognitive, emotional, and behavioral orientation toward the consumption or avoidance of animal-based foods, including red meat, poultry, fish, eggs, and dairy. This study aimed to adapt and validate the Dietarian Identity Questionnaire (DIQ) for Turkish-speaking populations by establishing its cultural and linguistic suitability and examining dietarian identity profiles among different dietary patterns. The DIQ was adapted into Turkish and administered via a web-based survey to 487 Turkish-speaking adults (Mean age = 28.9 ± 10.7 years; 34.7% male, 64.1% female, and 1.2% non-binary). Participants were categorized as omnivores, vegetarians, or vegans based on self-reported dietary exclusions. Structural validity was assessed using Confirmatory Factor Analysis, while internal consistency, composite reliability, and convergent and discriminant validity were evaluated through Cronbach’s alpha and average variance extracted. Group differences across dietary identity profiles were examined using ANOVA, and interrelationships among the DIQ-Turkish (DIQ-T) subscales were explored through Pearson correlation analysis. The results indicated that the eight-factor DIQ-T demonstrated a strong model fit (χ2 (465) =1841.45, p < 0.001, CFI = 0.924, TLI = 0.914, RMSEA = 0.078, SRMR = 0.050), confirming its structural validity. Reliability analysis indicated high internal consistency across all subscales, and significant differences were observed between omnivores and vegan/vegetarian groups across multiple dimensions (p < 0.001), highlighting distinct psychological and motivational patterns associated with dietarian identity. These findings support DIQ-T as a valid and reliable instrument for assessing dietarian identity, providing a valuable tool for researchers and health professionals investigating dietary behaviors and their potential implications for public health and nutrition interventions.

## Introduction

Food choices and dietary habits are deeply intertwined with cultural, social, and personal identities, reflecting individual preferences, beliefs, and values [1,2]. Understanding the intricate relationship between diet and identity has become a focal point in various fields, including psychology, sociology, and nutrition science. As individuals navigate the complex landscape of food culture, their dietarian identities emerge as significant components of their overall self-concept [3,4]. In various nations worldwide, including both vegetarians and vegans, individuals abstain from meat—a decision that transcends mere ethical or health considerations and constitutes a central aspect of their identity, profoundly influencing their social experiences and psychological well-being [5–7].

Until recently, studies have predominantly focused on disparities in environmental and health-related perceptions and attitudes between omnivores (individuals consuming all animal-derived foods such as meat, dairy, and eggs) and vegetarians, as well as vegans [8–10]. However, recent research suggests significant differences between omnivores and vegans/vegetarians not only in their dietary patterns but also in the motivations underlying their dietary choices and their relationships with other behaviors [11,12]. Understanding the variability in individuals’ motivations for adopting specific dietary patterns and food choices facilitates novel perspectives on the social cognition surrounding food, a pervasive stimulus encountered in daily interactions. Furthermore, examining the social, cultural, and moral values underpinning dietary behaviors towards animal-based or plant-based diets aids in comprehending how such values relate to everyday behaviors and attitudes [11]. Given the favorable effects of plant-based diets not only in mitigating climate change but also in promoting overall health and sustainability, understanding these values can inform the development of dietary recommendations and educational strategies [13,14].

Several instruments have previously been developed to assess the psychological and motivational dimensions of food choices and dietary practices [15–18]. These include the Food Choice Questionnaire, which focuses on motivations behind food selection [15]; the Eating Motivation Survey, which explores various motives and reasons underlying eating behavior [16]; the Health and Taste Attitude Scales, which assess orientations toward the health and hedonic characteristics of foods [17]; and the Meaning of Food in Life Questionnaire, which captures the personal significance of food across moral, sacred, health, social, and aesthetic dimensions [18]. In contrast to these tools, the Dietarian Identity Questionnaire (DIQ) offers a more comprehensive and multidimensional framework—encompassing not only behavioral but also affective, cognitive, social, and moral aspects of diet-related identity—and serves as a valuable instrument for assessing individuals’ identification with their dietary patterns and behaviors [12].

Dietarian identity encompasses self-perceptions, thoughts, feelings, and behaviors related to food selection and dietary behaviors, reflecting individuals’ personal and social components [12]. The DIQ provides insights into the psychological aspects of dietarian identity, exploring how individuals perceive themselves in relation to their food preferences and consumption patterns. Researchers have initiated inquiries into the factors distinguishing individuals opting for a plant-based diet from those consuming meat, exploring the underlying motivations driving such divergent dietary preferences, and examining the implications of one’s dietary choices for their sense of identity [19]. The DIQ demonstrated satisfactory psychometric properties, including internal consistencies, factor structure, construct validity, test–retest reliability, and replicability, among United States individuals following various dietary patterns [12]. Furthermore, validation of the DIQ within a strictly vegetarian sample also revealed robust psychometric properties [5]. The DIQ has been adapted and validated across a variety of cultural contexts, including English-, German-, Spanish-, Italian-, Brazilian-, and Saudi Arabian-speaking populations, and in all adaptations, the original eight-factor structure was consistently replicated [20–24].

The DIQ demonstrates potential as a valuable instrument for probing the psychology of eating behavior. Nevertheless, its usage has predominantly been observed within Western societies [20–22], while studies exploring its applicability in Eastern cultures and various other cultural contexts remain limited [23]. While the DIQ has been widely utilized in various cultural contexts, its adaptation and validation within specific cultural settings, such as the Turkish context, are essential to ensure its psychometric robustness and cross-cultural applicability. In recent years, Türkiye has experienced significant shifts in dietary patterns due to factors such as globalization, urbanization, and socioeconomic changes, sparking a growing interest in understanding Turkish individuals’ dietarian identities and their impact on health and well-being. However, despite the importance of dietarian identity in Türkiye, there is a notable lack of validated instruments for effectively assessing this construct. Therefore, this study aims to address this gap by adapting and validating the DIQ for use in Turkish populations through the assessment of its factor structure, internal consistency, composite reliability, and convergent and discriminant validity. Adaptation of the DIQ to the Turkish context holds promise for advancing our understanding of dietary behaviors and their implications for health promotion and intervention strategies in Türkiye and in other Turkish-speaking nations.

## Materials and methods

### Dietarian identity questionnaire

The DIQ is a self-report instrument originally developed and validated in English by Rosenfeld and Burrow [12]. The questionnaire begins with a screening question regarding animal product restrictions to categorize respondents’ dietary patterns, specifically identifying which animal products they typically abstain from consuming. The DIQ scale encompasses eight distinct dimensions, measured through multi-item subscales: centrality; private, public, and outgroup regard; prosocial, personal, and moral motivation; and strictness. These dimensions collectively reflect the degree to which individuals identify themselves based on their diets—either positively or negatively—evaluate others who follow different dietary patterns, feel motivated to maintain their dietary habits, and adhere to those habits. The DIQ consists of 33 items, each rated on a 7-point Likert scale ranging from 1 (“strongly disagree”) to 7 (“strongly agree”) [12].

### Translation procedures for the questionnaire

To adapt the DIQ for Turkish-speaking populations, a rigorous translation process was implemented in accordance with established practices for questionnaire adaptation [25]. The procedure involved two independent forward translations: one conducted by a professional translator with expertise in academic and psychological measurement tools, and the other by a native Turkish speaker fluent in English and residing in an English-speaking country. These two versions were then systematically reviewed and reconciled by an expert panel, ensuring conceptual and linguistic equivalence while preserving the original meaning of the items. Following this reconciliation, the finalized Turkish version underwent pretesting with a sample of 20 participants to assess clarity, cultural appropriateness, and comprehension. Participants provided qualitative feedback, which informed minor refinements to enhance readability and interpretability.

### Study procedures and participant characteristics

To assess the psychometric properties of the DIQ-T, a web-based survey was administered to a sample of Turkish individuals. The survey comprised a structured questionnaire divided into three sections. Prior to commencement, participants received information regarding the research objectives, participation conditions, and data privacy. Participation eligibility required individuals to be between the age of 18–65. The first section of the survey prompted participants to specify any dietary exclusions from a list of options, including red meat, poultry, fish, eggs, and dairy products. Section two focused on the 33 items of the DIQ-T, where participants rated their agreement with statements using a 7-point Likert scale ranging from “strongly disagree” to “strongly agree” [12]. Section three encompassed inquiries concerning sociodemographic characteristics, allergies and medical conditions, and dietary pattern categorization (omnivorous, flexitarian, vegetarian, or vegan) of the participants. It was found that no participants adhered to dietary patterns such as flexitarians. Consequently, participants were categorized into three groups: vegan, vegetarian, and omnivorous.

The sample size was determined based on established psychometric guidelines, which recommend five to ten participants per item. To ensure robust reliability and validity assessments, a total of 516 participants were recruited between March 2024 and May 2024, meeting the criteria for both exploratory and confirmatory factor analyses while enhancing the generalizability of the findings. However, subsequent examination of the dataset led to the exclusion of responses from participants who exhibited rapid completion, uniform responses, or reported dietary exclusions due to allergies or medical conditions [26]. Consequently, the final sample comprised 487 individuals. The sample had a mean age of 28.9 ± 10.7 years and was predominantly female (64.1%), with over half holding a bachelor’s degree (56.1%). The characteristics of the study population are given in Table 1.

**Table 1:** Characteristics of the study sample.

Characteristics	Frequency	Sample (%)
**Gender**		
** Male**	169	34.7
** Female**	312	64.1
** Nonbinary**	6	1.2
**Education**		
** Middle school**	11	2.3
** High school**	203	41.7
** Bachelor’s degree**	228	46.8
** Masters**	34	7
** PhD**	11	2.3
**Occupation**		
** Unemployed/Housewife**	29	6
** Student**	221	45.4
** Employee**	177	36.4
** Self-employed**	41	8.4
** Retired**	19	3.9

The research was conducted in compliance with the principles of the Declaration of Helsinki and was approved by Hacettepe University Social Sciences and Humanities Researches Ethics Board (Number of protocol: E-68552689-000-00003299295). Written informed consent was obtained from all participants prior to their involvement in the research.

### Statistical analysis

A confirmatory factor analysis (CFA) was conducted with IBM SPSS AMOS v23 to validate the DIQ-T. Within this CFA, the DIQ model was determined in the same way as Rosenfeld and Burrow [12]. The model tested included eight distinct latent variables, which were the eight factors of the DIQ: ‘(1) centrality, (2) private regard, (3) public regard, (4) outgroup regard, (5) prosocial motivation, (6) personal motivation, (7) moral motivation, and (8) strictness’. According to the CFA results, various indices were used to evaluate the fit of the model. Although χ2 is commonly reported in CFA research, other fit indices are typically given greater emphasis in assessing model fit. According to established methodological guidelines [27–31], model fit was evaluated by the comparative fit index (CFI), the Tucker–Lewis index (TLI), the root-mean-square-error of approximation (RMSEA), and the standardized root-mean-square residual (SRMR). Interpretation criteria were derived from the referenced methodological standards, acceptable and excellent model fit are indicated by CFI and TLI values greater than or close to 0.90–0.95, and by RMSEA and SRMR values smaller than or close to 0.08–0.06.

The validation of the model as a result of CFA explains that the factors in the scale and the items belonging to these factors have a certain level of convergent and discriminant validity. However, it is still stated that additional evidence regarding convergent and discriminant validity can be presented. Composite reliability (CR) and average variance extract (AVE) values were used to evaluate the convergent and discriminant validity of the DIQ-T scale [32]. If the values of CR and AVE for each factor exceed 0.70 and 0.50, respectively, the scale is considered having convergent and discriminant validity [33].

Cronbach’s alpha was used to assess the internal consistency of the DIQ-T. Skewness and kurtosis coefficients, histogram graphs and Kolmogorov-Smirnov/Shapiro-Wilk tests were used to analyse whether the variables were normally distributed. Since the variables were normally distributed, ANOVA test was used to compare the groups. DIQ-T subscale scores of omnivores, vegans, and vegetarians were evaluated with ANOVA. Correlations among the DIQ-T subscales were analyzed using Pearson correlation. A statistical significance level of p < 0.05 was considered acceptable.

## Results

The distribution of participants among omnivores, vegans and vegetarians is summarized in Table 2. The mean age of the omnivores (n = 370) was 28.93 ± 11.26 years, vegans (n = 50) 29.92 ± 8.88 years, and vegetarians (n = 67) 28.01 ± 8.97 years (p = 0.636).

**Table 2:** Distribution of participants among diet groups.

	Omnivores (n = 370)		Vegans (n = 50)		Vegetarians (n = 67)	
	**n**	**%**	**n**	**%**	**n**	**%**
**Gender**						
** Female**	227	72.8	37	11.9	48	15.4
** Male**	142	84.0	11	6.5	16	9.5
** Nonbinary**	1	16.7	2	33.3	3	50.0
**Age (Mean±SD)**	28.93 ± 11.26		29.92 ± 8.88		28.01 ± 8.97	

Table 3 presents the mean values, standard deviations, Cronbach’s α, CR, and AVE for the DIQ-T. The internal consistency of the subscales was high, as indicated by Cronbach’s α values ranging from 0.85 to 0.96. The Centrality subscale had a mean score of 5.00 ± 1.70, with a Cronbach’s α of 0.96, CR of 0.96, and AVE of 0.84. The Private Regard and Public Regard subscales exhibited similar mean scores (4.19 ± 1.90 and 4.26 ± 2.01, respectively), both with high internal consistency (α = 0.90 and α = 0.92) and AVE values above 0.75, supporting the convergent validity of these constructs. The Outgroup Regard subscale had a mean score of 5.19 ± 1.83, Cronbach’s α of 0.96, CR of 0.96, and AVE of 0.79. Regarding motivational dimensions, Prosocial Motivation had a mean score of 3.85 ± 2.02 with excellent reliability (α = 0.95) and an AVE of 0.78, while Personal Motivation demonstrated strong internal consistency (α = 0.88) with an AVE of 0.71, supporting its measurement stability. Moral Motivation, which had the lowest mean score (3.38 ± 2.01), exhibited good reliability (α = 0.85) with an AVE of 0.67, indicating an adequate representation of the construct. Finally, Strictness, with a mean score of 3.90 ± 2.05, demonstrated high reliability (α = 0.90, CR = 0.90) and an AVE of 0.76, further supporting the validity and consistency of the scale.

**Table 3:** Mean scale values, standard deviations, Cronbach’s α, composite reliability and average variance extract.

DIQ-T Scale	Mean±SD	Cronbach’s alpha	CR	AVE
**Centrality (5 items)**	5.00** ± **1.70	0.96	0.96	0.84
**Private Regard (3 items)**	4.19** ± **1.90	0.90	0.90	0.76
**Public Regard (3 items)**	4.26** ± **2.01	0.92	0.92	0.80
**Out-group Regard (7 items)**	5.19** ± **1.83	0.96	0.96	0.79
**Prosocial Motivation (6 items)**	3.85** ± **2.02	0.95	0.95	0.78
**Personal Motivation (3 items)**	4.41** ± **1.88	0.88	0.88	0.71
**Moral Motivation (3 items)**	3.38** ± **2.01	0.85	0.85	0.67
**Strictness (3 items)**	3.90** ± **2.05	0.90	0.90	0.76

DIQ-T; Dietarian Identity Questionnaire- Turkish, CR; composite reliability, AVE; average variance extract.

**Fig 1:**
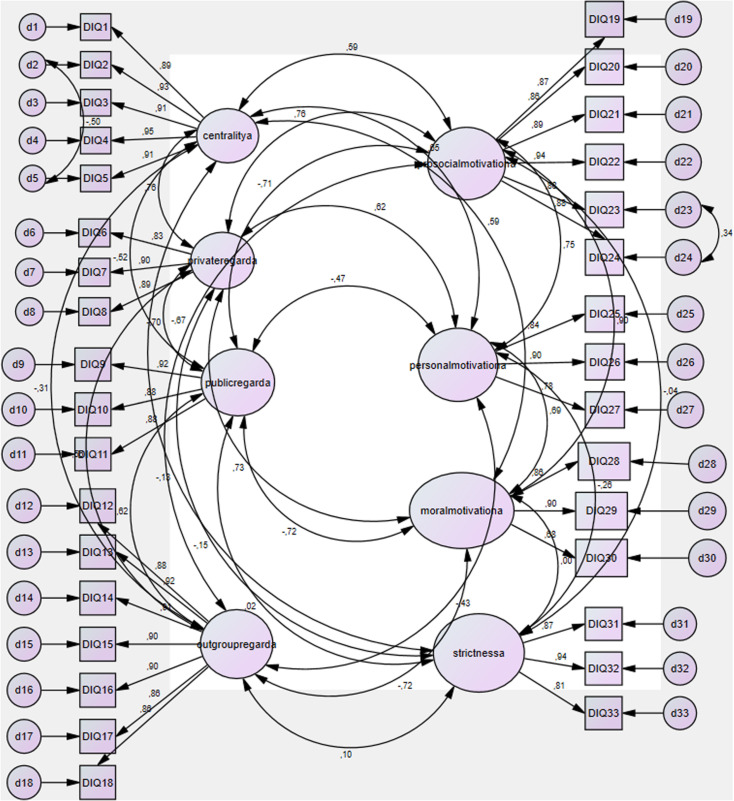
Path diagram of the confirmatory factor analysis (CFA) for the Turkish version of the Dietarian Identity Questionnaire (DIQ-T). The figure illustrates the eight-factor structure of the DIQ-T based on confirmatory factor analysis. Each latent variable corresponds to a subscale of the DIQ-T: centrality, private regard, public regard, outgroup regard, prosocial motivation, personal motivation, moral motivation, and strictness.

When the CFA was conducted on the full sample, the eight-factor DIQ-T model demonstrated a good fit to the data: χ2 (465) = 1841.45, p < 0.001, CFI = 0.924, TLI = 0.914, RMSEA = 0.078, SRMR = 0.050. The factor loadings of the scale items derived from the CFA are presented in the path diagram. An examination of the standardized parameter estimates in Fig 1 reveals that the factor loadings range from 0.68 to 0.95. All loadings were statistically significant (p < 0.001) and exceeded the conventional cutoff value of 0.30, indicating strong associations between observed variables and their respective latent constructs. Additionally, AVE per construct was higher than 0.50, and CF per construct was higher than the conventional cutoff of 0.70. Internal consistencies were sufficiently high for all eight DIQ factors, ranging from α = 0.85 for moral motivation to α = 0.96 for centrality.

**Fig 2:**
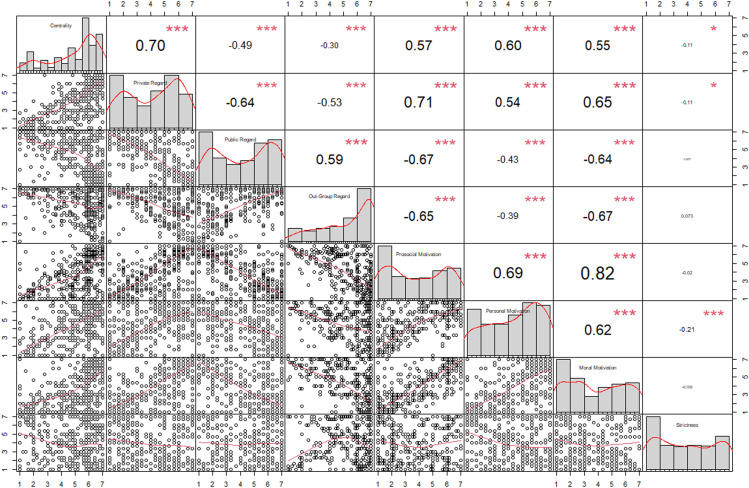
Correlations among the DIQ-T subscales. The figure displays pairwise scatterplots, histograms, and Pearson correlation coefficients among the DIQ-T subscales. * p < 0.05, *** p < 0.001.

Fig 2 illustrates the Pearson’s r correlation coefficients among the DIQ-T subscales. Significant positive correlations were observed between Prosocial Motivation and Personal Motivation (r = 0.82, p < 0.001), as well as between Centrality and Personal Motivation (r = 0.60, p < 0.001), suggesting strong associations between these constructs. Similarly, Centrality showed a positive correlation with Prosocial Motivation (r = 0.57, p < 0.001). Conversely, Public Regard was negatively correlated with Outgroup Regard (r = −0.65, p < 0.001) and Prosocial Motivation (r = −0.67, p < 0.001), indicating that individuals with higher public regard scores tend to have lower prosocial and outgroup regard tendencies.

Table 4 shows the differences in DIQ-T scores among omnivores, vegans, and vegetarians. Statistically significant differences were observed across all dimensions of the DIQ-T (p < 0.001). Vegans and vegetarians exhibited significantly higher scores than omnivores in Centrality, indicating a stronger identification with their dietary choices. Similarly, Private Regard was significantly greater in vegans and vegetarians compared to omnivores, suggesting that individuals following plant-based diets have a more positive internal evaluation of their dietarian identity. Conversely, Public Regard scores were significantly lower in vegans and vegetarians than in omnivores, implying that plant-based eaters perceive lower societal recognition or approval of their dietary choices. Omnivores reported significantly higher scores on Outgroup Regard compared to vegans and vegetarians, suggesting that they may hold more favorable perceptions of individuals outside their dietary group. In terms of motivation, both Prosocial Motivation and Moral Motivation were significantly higher in vegans and vegetarians compared to omnivores, highlighting ethical and altruistic concerns as strong drivers for plant-based eaters. Similarly, Personal Motivation was significantly higher among vegans and vegetarians than omnivores, suggesting greater intrinsic commitment to their dietary patterns. Lastly, Strictness was markedly higher in vegans and vegetarians compared to omnivores, reflecting a more rigid adherence to dietary rules among plant-based eaters. Post-hoc comparisons indicated that omnivores significantly differed from both vegans and vegetarians across all variables, while no significant differences were found between vegans and vegetarians, except for Centrality, where vegetarians scored higher. These findings suggest that individuals following plant-based diets exhibit stronger dietarian identification, higher moral and prosocial motivations, and greater dietary adherence compared to omnivores.

**Table 4:** Differences in DIQ-T subscale scores among omnivores, vegetarians, and vegans.

	Omnivores (n = 370) Mean±SD	Vegans (n = 50) Mean±SD	Vegetarians (n = 67) Mean±SD	p-value
**Centrality**	4.68 ± 1.75^a^	5.92 ± 1.34^b^	6.18 ± 0.58^b^	<0.001
**Private Regard**	3.81 ± 1.87^a^	5.30 ± 1.72^b^	5.50 ± 1.24^b^	<0.001
**Public Regard**	4.72 ± 1.89^a^	2.81 ± 1.95^b^	2.82 ± 1.51^b^	<0.001
**Out-group Regard**	5.41 ± 1.84^a^	4.65 ± 1.63^b^	4.38 ± 1.63^b^	<0.001
**Prosocial Motivation**	3.28 ± 1.85^a^	5.53 ± 1.60^b^	5.78 ± 1.22^b^	<0.001
**Personal Motivation**	4.12 ± 1.92^a^	5.32 ± 1.51^b^	5.38 ± 1.33^b^	<0.001
**Moral Motivation**	3.36 ± 1.86^a^	5.41 ± 1.65^b^	5.38 ± 1.71^b^	<0.001
**Strictness**	3.36 ± 1.87^a^	5.77 ± 1.47^b^	5.56 ± 1.62^b^	<0.001

Values are presented as mean ± standard deviation. Group differences were assessed using ANOVA. Superscript letters within each row (a, b) indicate significant pairwise differences between groups based on post-hoc comparisons.

## Discussion

This study represents the first adaptation and validation of the DIQ for Turkish-speaking populations. The results indicate strong internal consistency and reliability across the different subscales of the DIQ-T Scale. These results suggest significant associations between the DIQ-T subscales, further supporting the scale’s construct validity. Hence, the eight-factor structure of the DIQ, previously identified in studies with English [12], Italian [20], German [21], Spanish [22] and Portuguese-speaking [24] populations, was also confirmed in the Turkish sample. This replication showed a strong model fit and consistency with the structure observed in prior studies, despite cultural variations.

The observed significant correlations among the DIQ-T subscales provide valuable insights into the interrelationships between different dimensions of dietarian identity. The strong associations between centrality, private regard, and motivational factors suggest that individuals with a stronger identification with their dietary choices and dietary pattern also exhibit higher personal and moral motivations for adherence. These findings are consistent with existing literature which emphasizes the interplay between self-perception, external validation, moral and intrinsic motivation in shaping dietary behaviors [20–24].

The subscales of DIQ were found to differ significantly among individuals adhering to different dietary patterns, particularly between vegans/vegetarians and omnivores. These findings align with previous studies conducted in other cultural contexts [20–22], further supporting the cross-cultural validity of the construct. In the present study, those following a vegan or vegetarian diet scored higher in centrality, private regard, prosocial motivation, personal motivation, moral motivation, and strictness, while scoring lower in public regard and out-group regard compared to omnivores. Similar trends in the same subscales were reported in German [21], Italian [20], and Spanish [22] populations. Specifically, higher centrality scores indicate that diet plays a more pivotal role in shaping their self-concept of vegan and vegetarians compared to omnivores. Vegan and vegetarians also demonstrated higher private regard scores, suggesting stronger personal validation and satisfaction with their dietary choices, underscoring a deep personal connection to their diet. Moreover, their higher scores in prosocial motivation reflect a stronger inclination towards dietary choices that benefit others or align with ethical considerations, which was notably pronounced compared to omnivores. Additionally, vegan and vegetarians showed higher personal motivation scores, indicating a strong internal drive and personal reasons underpinning their dietary practices. This was complemented by higher moral motivation scores, indicating a heightened sense of ethical obligation or moral reasoning guiding their dietary decisions, a trend significantly higher than observed in omnivores. Similarly, their higher scores in strictness underscored a greater adherence to dietary rules and guidelines compared to omnivores. Conversely, participants scored lower in public regard, indicating less concern for societal perceptions of their diet, and lower out-group regard scores, suggesting less positive regard towards individuals or groups with differing dietary practices. This distinction highlights a preference among participants for aligning their diet with personal values rather than seeking external validation or conforming to societal norms. These findings underscore a robust pattern of dietarian identity among vegans and vegetarians characterized by strong personal commitment, ethical considerations, and a focus on personal values rather than external validation or societal norms. This alignment across multiple dimensions of dietarian identity supports the notion of a cohesive and internally consistent dietary identity among those following these diets, despite cultural and social differences [20–22]. Notably in the present study, DIQ subscales’ scores did not differ statistically between vegans and vegetarians (p > 0.05) similar to the Italian population [20]. Conversely, a prior study highlighted that vegans, compared to vegetarians, perceived their dietary choices as more central to their personal identity while they also expressed higher levels of personal satisfaction and value within their dietary community, reflecting increased private regard. Additionally, vegans exhibited stronger motivations for adhering to their dietary habits, including enhanced prosocial, personal, and moral motivations [4]. These discrepancies might be influenced by variations in sample characteristics and cultural contexts across studies, shaping the observed differences in dietarian identity profiles between vegans and vegetarians.

### Theoretical implications

The DIQ serves as a crucial instrument for gaining a deeper and more nuanced understanding of dietarian identities and behaviors. Exploring the fundamental psychological and social drivers behind the adoption of vegetarian or vegan diets offers valuable insight into how individuals relate to food on an identity level [5,34]. The availability of a culturally and linguistically validated version of the DIQ enables researchers to accurately capture these identity dynamics within the Turkish context, thereby facilitating reliable data collection and contributing to cross-cultural research in nutritional psychology and identity theory. Moreover, by confirming the multidimensional structure of the DIQ in a Turkish sample, this study supports the generalizability of diet-related identity constructs beyond Western populations.

### Practical implications

In the field of health and nutrition, the validated DIQ-T may support the identification of dietary patterns and their implications for health outcomes, thereby informing targeted interventions and personalized dietary strategies. From a public health perspective, understanding population-level dietarian identities can aid in the development of policies and health promotion initiatives aimed at improving dietary behaviors and reducing nutrition-related diseases. Additionally, in the field of nutrition and dietetics, the DIQ-T provides practitioners with a valuable tool to assess and support individuals in their dietary choices, ultimately enhancing the effectiveness of dietary counselling and education programs.

### Strengths and limitations

This study has several notable strengths. First, it is the first adaptation and validation of the DIQ in a Turkish-speaking population, ensuring its linguistic and cultural suitability. Additionally, the study offers valuable insights into the psychological and motivational dimensions of dietarian identity, highlighting significant differences between omnivores, vegetarians, and vegans. However, it is important to acknowledge several limitations of the study. First, a limitation arises from the study sample’s representativeness. While the percentages offer a reasonably accurate depiction of the diversity of dietarian identities, the sample shows a lack of representation, particularly among vegetarians and vegans. Thus, future studies could enhance results pertaining to these dietary groups by examining samples consisting solely of vegans and/or vegetarians. Secondly, a limitation is associated with the reliance on a self-reported instrument, which could be influenced by social desirability bias. Therefore, it is advisable for future studies to thoroughly investigate whether there is an intermediary relationship between self-categorization and an indirect assessment of diet-related profiles.

## Conclusion

This study contributes to the growing body of literature on dietarian identity by examining distinctions among omnivores, vegetarians, and vegans, while also expanding cross-cultural validation efforts of the DIQ. The successful adaptation and validation of the DIQ-T represent a significant advancement in assessing dietarian identity within the Turkish context. The findings confirm the strong psychometric properties of the DIQ-T, demonstrating its reliability, validity, and sensitivity across different dietary groups. The distinct differences observed among omnivores, vegetarians, and vegans highlight the DIQ-T’s ability to capture the nuanced psychological and motivational dimensions of dietarian identity. This validated instrument offers a valuable tool for researchers and practitioners seeking to explore dietary behavior, dietary choices and their implications for health and well-being. By accurately identifying dietarian profiles and their underlying motivations, the DIQ-T can inform targeted public health interventions and contribute to evidence-based strategies promoting healthier and more sustainable dietary choices. Future studies could explore longitudinal applications, cross-cultural comparisons, and complementary assessment methods to further enhance the understanding of dietarian identity and its broader implications, while also utilizing this instrument to gain deeper insights into dietary behaviors, social influences, and health-related outcomes across diverse populations.

## Supporting information

S1 File The Questionnaire. This file contains the Turkish version of the Dietarian Identity Questionnaire (DIQ) used in the study, along with its English translation.
(PDF)

S2 File Raw data. This file includes the raw dataset generated and analyzed during the current study.
(XLSX)
