# Supplementary material for: Validation and adaptation of a Turkish version of the dietarian identity questionnaire
Source: PLoS One. 2025 Jun 25;20(6):e0327116. doi: 10.1371/journal.pone.0327116 (PMC12193050; doi:10.1371/journal.pone.0327116)
Supplement: S1 File — This file contains the Turkish version of the Dietarian Identity Questionnaire (DIQ) used in the study, along with its English translation. (PDF) [file pone.0327116.s001.pdf]

SUPPLEMENTARY INFORMATION -1

The Questionnaire – Turkish (Original)

Beslenme Kimliği Anketi – Türkçe (BKA-T)

I. Bölüm

Beslenme örüntüsü:

Genel olarak, aşağıdaki besin gruplarından hangilerini tüketmezsiniz? Lütfen tüketmediğiniz besin gruplarını seçin. Eğer genellikle bu besin gruplarının hepsini tüketiyorsanız, lütfen son cevabı seçin.

a. Genellikle kırmızı et tüketmem
b. Genellikle kümes hayvanlarını tüketmem
c. Genellikle balık tüketmem
d. Genellikle süt ürünleri tüketmem
e. Genellikle yumurta tüketmem
f. Genellikle bu besin gruplarının hepsini tüketirim

Bu anketin geri kalanında lütfen "beslenme örüntünüz"ün yukarıda belirttiğiniz besinleri temsil ettiğini unutmayın. Örneğin, "kırmızı et" ve "süt ürünleri" seçtiyseniz, beslenme örüntünüz kırmızı eti ve süt ürünlerini içermez. Eğer son cevabı seçtiyseniz, beslenme örüntünüz tüm bu besinleri içerir.

II. Bölüm

Lütfen aşağıdaki ifadelerin her birine hangi düzeyde katıldığınızı veya katılmadığınızı belirtiniz.

	Kesinlikle katılmıyorum	Katılmıyorum	Çok az katılmıyorum	Kararsızım	Çok az katılıyorum	Katılıyorum	Kesinlikle katılıyorum
1. Beslenme örüntüm, kendimi tanımlamamın önemli bir parçasıdır.							
2. Beslenme örüntüm kendim hakkında nasıl düşündüğüm üzerinde büyük bir etkiye sahiptir.							
3. Yaşam tarzımın büyük bir kısmı, beslenme örüntüm etrafında döner.							
4. Beslenme örüntüm kim olduğumun önemli bir yönünü tanımlar.							
5. Beslenme örüntüme uymak kim olduğumun önemli bir parçasıdır.							
6. Benimle aynı beslenme örüntüsüne sahip kişiler, genellikle iyi insanlardır.							
7.Beslenme örüntüme uymak saygın bir yaşam tarzıdır.							
8.Beslenme örüntüme uyan insanlar, besin seçimlerinden gurur duymalıdır							
9.Beslenme örüntüme uyan insanlar, besin seçimleri konusunda olumsuz yargılanır.							

10.Beslenme örüntüme uyan insanlar, besin seçimleri konusunda eleştiri alabilirler.							
11.Beslenme örüntüme uymak, olumsuz kalıp yargılarla ilişkilidir.							
12.Beslenme örüntüme aykırı olan besinleri tüketen insanları daha az ahlaklı bulurum.							
13.Beslenme örüntüme aykırı besinleri tükettikleri için insanları olumsuz yargılarım.							
14.Beslenme örüntüme aykırı besinleri tüketen insanları görmek beni üzer veya sinirlendirir.							
15.Eğer benim beslenme örüntüme aykırı besinleri tüketen birini görürsem, onu daha az severim.							
16.İnsanların beslenme örüntüme aykırı besinleri tüketmesi beni rahatsız eder.							
17.Beslenme örüntüme aykırı besinleri tüketen birini görmek, onu benim için daha az çekici hale getirir.							
18. İnsanlar benim beslenme örüntüme aykırı olan besinleri tükettikleri için kendilerini suçlu hissetmelidir.							
19.Beslenme örüntümü dünyayı diğerleri için daha iyi bir yer haline getirmenin bir yolu olarak görüyorum.							
20. Sosyal sorunlar, beslenme örüntüme uymam konusunda beni motive ediyor.							
21. Topluma fayda sağlamak istediğim için beslenme örüntüme uyuyorum.							
22. Besin seçimlerimin diğer varlıklar üzerindeki etkilerinden endişe duyduğum için beslenme örüntüme uyma konusunda kendimi motive hissediyorum.							
23. Başkalarına yardım etmek istediğim için beslenme örüntüme uymaya motiveyim.							
24. Kendi beslenme örüntüme uyuyorum çünkü bu şekilde beslenmek dünya için iyidir.							
25. Besin seçimlerimin kendi sağlığım üzerindeki etkilerinden endişe duyduğum için beslenme örüntüme uyuyorum.							
26.Beslenme örüntüme, bu şekilde beslenmek hayatımı geliştirdiği için uyuyorum.							
27. Hangi hayvansal ürünleri tüketeceğimi düşünürken besin seçimlerimin kendi sağlığım üzerindeki etkilerini göz önünde bulundururum.							
28.Beslenme örüntüme uyma konusunda ahlaki bir sorumluluğum olduğunu hissediyorum.							
29. Beslenme örüntüme uymaya motiveyim çünkü beslenme örüntüme aykırı olan besinleri tüketmek ahlaka aykırıdır.							
30. Beslenme örüntüme uyuyorum çünkü bu şekilde beslenmek ahlaki açıdan doğrudur.							
31.Bazen esnek olabilir ve beslenme örüntüme aykırı olan besinleri tüketebilirim.							
32. Zaman zaman beslenme örüntüme aykırı besinleri tüketirim.							
33. Eğer bir yiyeceğin tadının olağanüstü iyi olduğunu duysaydım, beslenme örüntüme aykırı olmasına rağmen onu tüketirdim.							

III. Bölüm
34. Cinsiyetiniz:

35. Yaşınız:

36. Eğitim düzeyiniz:
a.İlköğretim
b.Lise
c. Lisans
d. Yüksek lisans
e. Doktora

37. Mesleğiniz:
a. İşsiz
b. Ev hanımı
c. İşçi/ Memur
d. Serbest meslek
e. Emekli
f. Öğrenci

38. Herhangi bir alerjiniz var ise belirtiniz:

39. Doktor tarafından tanısı konmuş herhangi bir hastalığınız var ise belirtiniz:

40. Beslenme şeklinizi nasıl tanımlarsınız:
a. Omnivor
b. Fleksetaryen
c. Pesketaryen
d. Vejetaryen
e. Vegan
f. Bilmiyorum / Fikrim yok

The Questionnaire (English Translation)

I. Section

The Dietarian Identity Questionnaire (DIQ) (English version)
Dietary pattern:
In general, which of the following food groups do you not eat? Please select all that apply. If you generally eat all of these food groups, please select the last response.

- I generally do not eat red meat
- I generally do not eat poultry
- I generally do not eat fish
- I generally do not eat dairy
- I generally do not eat egg
- I generally eat all of these food groups

For the rest of this survey, please note that your "dietary pattern" represents those foods you indicated above. For example, if you selected "red meat" and "dairy," your dietary pattern excludes red meat and dairy. If you selected the last response, your dietary pattern includes all of these foods.

II. Section

Please indicate how strongly you agree or disagree with each of the following statements.

	Strongly disagree	Disagree	Somewhat disagree	Neither agree nor disagree	Somewhat agree	Agree	Strongly agree
1. My dietary pattern is an important part of how I would describe myself.							
2. My dietary pattern has a big impact on how I think of myself.							
3. A big part of my lifestyle revolves around my dietary pattern.							
4. My dietary pattern defines a significant aspect of who I am.							
5. Following my dietary pattern is an important part of who I am.							
6. People who follow my dietary pattern tend to be good people.							
7. Following my dietary pattern is a respectable way of living.							
8. People who follow my dietary pattern should take pride in their food choices.							
9. People who follow my dietary pattern are judged negatively for their food choices.							
10. People who follow my dietary pattern tend to							

receive criticism for their food choices.							
11. Following my dietary pattern is associated with negative stereotypes.							
12. I view people as less moral for eating foods that go against my dietary pattern.							
13. I judge people negatively for eating foods that go against my dietary pattern.							
14. Seeing people eat foods that go against my dietary pattern makes me upset or angry.							
15.If I see someone eat foods that go against my dietary pattern, I like him or her less							
16.It bothers me when people eat foods that go against my dietary pattern							
17. Seeing someone eat foods that go against my dietary pattern makes him or her less attractive to me.							
18. People should feel guilty about eating foods that go against my dietary pattern.							
19. I view my dietary pattern as a way of making the world a better place for others.							
20. Concerns about social issues motivate me to follow my dietary pattern.							
21.I follow my dietary pattern because I want to benefit society.							
22. I feel motivated to follow my dietary pattern because I am concerned about the effects of my food choices on other beings.							
23. I am motivated to follow my dietary pattern because I want to help others.							
24. I follow my dietary pattern because eating this way is good for the							

world.							
25. I follow my dietary pattern because I am concerned about the effects of my food choices on my own well-being.							
26. I follow my dietary pattern because eating this way improves my life.							
27. When thinking about which animal products to consume, I consider the effects of my food choices on my own health.							
28. I feel that I have a moral obligation to follow my dietary pattern.							
29. I am motivated to follow my dietary pattern because eating foods that go against my dietary pattern is immoral.							
30. I follow my dietary pattern because eating this way is the morally right thing to do.							
31. I can be flexible and sometimes eat foods that go against my dietary pattern.							
32. From time to time, I eat foods that go against my dietary pattern.							
33. I would eat a food product that goes against my dietary pattern if I were to hear that it tastes exceptionally good.							

III. Section

34. Your Gender:

35.Your Age:

36. Your Educational Level:
   a. Primary education
   b. High school
   c. Bachelor's degree

d. Master's degree
e. Doctorate

37. Your Occupation:
a. Unemployed
b. Housewife
c. Worker
d. Self-employed
e. Retired
f. Student

38. If you have any allergies, please specify:
39. If you have been diagnosed with any medical condition by a doctor, please specify:
40. How would you define your dietary pattern?
   a. Omnivore
   b. Flexitarian
   c. Pescatarian
   d. Vegetarian
   e. Vegan
   f. I don’t know / No opinion
